# Production of monoclonal antibodies (mAbs) against oralcarcinoma & its bioconjugation with solid lipid nanoparticles (SLN) for drug delivery

**DOI:** 10.1007/s12672-026-05229-0

**Published:** 2026-05-26

**Authors:** Sanskruti Shrenik Patil, Kusuma Kumari Garikapati, Tallapaneni Vyshnavi, Abhishek Dey, Vasanth Raj Palanimuthu

**Affiliations:** 1 Department of Pharmaceutical Biotechnology, JSS College of Pharmacy, Ooty, The Nilgiris, Tamilnadu India; 2 Department of Pharmaceutical Biotechnology, Manipal College of Pharmaceutical Sciences, Manipal, Karnataka India; 3 Department of Pharmaceutics, JSS College of Pharmacy, Ooty, The Nilgiris, Tamilnadu India; 4 Department of Pharmacology, JSS College of Pharmacy, Ooty, The Nilgiris, Tamilnadu India; 5 School of Pharmacy, Queen’s University of Belfast, Belfast, BT9 7BL Northern Ireland, UK

## Abstract

This preliminary study explores a dual strategy of augmenting anticancer therapy with Carboplatin-loaded solid lipid nanoparticles (SLNs) alongside antigen-specific antibodies through immunization of KB oral carcinoma cells. KB and Sp2/01 myeloma cells were cultured and assessed for viability. Female Balb/c mice were immunized with KB cells, resulting in significant weight gainand spleen enlargement, suggesting immune activation. Enzyme-linked immunosorbent assay (ELISA) confirmed the production of antigen-specific antibodies, as indicated by an increase in absorbance at a 1:1000 serum dilution. Splenocytes from immunized mice were fused with myeloma cells using polyethylene glycol (PEG), with optimal hybridoma generation achieved in the third trial. SLNs were formulated by the microemulsion method, which exhibited favourable physicochemical properties (particle size: 144.57 nm, PDI: 0.261, zeta potential: -32 mV). Cytotoxicity assays revealed time- and dose-dependent cytotoxic effects against oral squamous cell carcinoma (OSCC), with SLNs demonstrating greater potency compared to free Carboplatin. These findings underscore the promise of SLNs in cancer therapy and the generation of KB cell-targeting antibodies using hybridoma technology.

**Supplementary Information:**

The online version contains supplementary material available at 10.1007/s12672-026-05229-0.

## Introduction

 Oral squamous cell carcinoma (OSCC) stands as one of the most significant burdens worldwide, currently ranking eighth in global incidence and representing one of the top three cancer types in the Indian population. OSCC predominantly affects men in their fifth to eighth decades of life [1]. However, emerging demographic studies report a rising incidence in adults, even among non-smoking, non-drinking women [2]. In India, tobacco is the primary risk factor for OSCC, though methods of consumption vary widely across regions. This evolving pattern complicates prevention strategies, as traditional risk factors such as tobacco and betel-quid chewing remain prevalent in India, but a notable proportion (15–20%) of OSCC cases occur without exposure to recognized carcinogens like tobacco or alcohol [3]. Notably, incidence in women aged over 70 years is increasing, especially at non-lingual sites with smaller tumour sizes [4]. The molecular underpinnings of OSCC in these patients remain underexplored, demanding innovative strategies in both diagnosis and therapy.

In response to this clinical challenge, cancer treatment modalities havecome a long way with targeted therapies that aim to halt tumor development by blocking key signalling pathways [5]. These therapies work by interfering with tumour-specific signalling pathways and include monoclonal antibodies (mAbs), small-molecule inhibitors, and immune checkpoint blockade, all of which have improved patient outcomes in selected head and neck cancers [6, 7]. Immunotherapy, an innovative treatment modality, utilizes biological response modifiers such as cytokines, vaccines, and genetically engineered monoclonal antibodies to facilitate the body’s own immune response against cancer [8].

The clinical impact of Monoclonal antibodies (mAbs) is magnified by ongoing advances in recombinant technology, which enables the creation of highly specific and potent antibodies [9, 10]. Advances in recombinant technology have led to the development of recombinant monoclonal antibodies that exhibit enhanced specificity and therapeutic effects compared to conventional monoclonal antibodies [11]. Conjugation techniques enable the production of antibody-drug conjugates (ADCs), where monoclonal antibodies are linked to potent chemotherapeutic drugs [12]. This targeted drug delivery strategy improves the precision of therapy, maximising tumor cell eradication while minimising damage to healthy tissues [13]. Importantly, the regulatory approval of several ADCs underscores their effectiveness across a spectrum of solid tumors, including malignancies of the oral cavity.

Alongside biologics, solid lipid nanoparticles (SLNs) have emerged as an innovative class of nanocarrier systems designed to stabilise drugs, increase solubility, and provide targeted delivery [14]. Compared to conventional drug formulations, SLNs exhibit biocompatibility, provide controlled drug release, and possess the ability to circumvent multidrug resistance mechanisms [15]. When combined with chemotherapeutics, SLNs contribute to improved tumour targeting and minimized systemic toxicity, making them a compelling option for greater efficacy, lower systemic toxicity, and improved patient outcomes [16]. Mechanistically, SLNs improve cellular uptake and help circumventefflux pumps that often contribute to chemotherapy resistance, making them highly promising for refractory cancers.

Bioconjugation techniques have further enhanced the efficacy of drugs by enabling the conjugation of monoclonal antibodies with nanoparticle-based drug delivery agents [17]. This approach facilitates precise targeting of cancer cells while minimising off-target effects. Antibody-drug conjugates (ADCs), constructed by linking monoclonal antibodies with cytotoxic payloads through stable linkers, have emerged as a highly effective therapeutic approach in cancer therapy [18].However, challenges such as complex manufacturing processes, potential immunogenicity, and the need for stable linker chemistries remain critical considerations in ADC development.

Situating the current investigation within this landscape, the present research aims to developmonoclonal antibodies against OSCC-specific antigen and conjugate them with the chemotherapeutic agent Carboplatin via advanced nanocarrier systems. Carboplatin is chosen due to its established efficacy in treating OSCC and its potential for enhanced therapeutic index when delivered specifically to cancer cells via antibody conjugation. This approach not only addresses ongoing clinical needs for improved therapeutic selectivity and reduced adverse effects but is also strongly aligned with recent translational trends in personalised cancer management. Through this strategy, new avenues for treating difficult-to-manage cases, particularly among non-traditional patient populations, may be realised.

## Materials and methodology

### Materials

The oral squamous cell carcinoma (OSCC) KB cell line and the myeloma Sp2/0 cell line were used to produce the antibodies (procured from the National Centre for Cell Sciences, Pune, India). The animals used for immunization were Balb/c mice, female, aged 6–8 weeks, weighing 10–15 g. The study received approval from the Institutional Animal Ethics Committee (JSSCP/OT/IAEC/21/2021-22) and the experiments were performed at JSS College of Pharmacy, Ooty.

### Maintenance of oral carcinoma cell line (KB cells)

KB cells were cultured in Minimum Essential Medium (MEM) supplemented with 10% Fetal Bovine Serum (FBS). The culture was incubated at 37 °C in a humidified atmosphere containing 5% CO₂. The culture medium was replaced every 48 h to ensure optimal nutrient levels and cell growth. Upon reaching approximately 80–90% confluency, the cells were subcultured at a ratio of 1:3 to sustain continuous to sustain continuous propagation and viability.

### Fluorescence assay

KB cells were seeded in six-well plates and cultured for 24 h under standard conditions (37 °C with 5% CO₂). After incubation, the cells were stained with an equal volume mixture (1:1) of acridine orange and ethidium bromide. Following staining, the cells were immediately observed under a fluorescence microscope to assess cellular viability and morphology [19].

### Immunization

Harvested KB cells were washed thoroughly with phosphate-buffered saline (PBS) and emulsified with Freund’s Complete Adjuvant. Female Balb/c mice (*n* = 6) received intraperitoneal injections of 0.4 mL of the emulsified antigen on days 0, 6, 13, 20, and 26. The body weight of the mice was monitored regularly to evaluate health status and tolerance to immunization [20].

### Antibody screening

Blood samples were collected from the immunized mice by cardiac puncture under anesthesia. The collected blood was allowed to clot at room temperature, followed by centrifugation to separate the serum, which was subsequently used for subsequent antibody screening assays [21].

### Enzyme-linked immunosorbent assay (ELISA) for antibody detection

KB cells were immobilized onto a 96-well microtiter plate via fixation with 0.05% glutaraldehyde. After washing, nonspecific binding sites were blocked by incubation with 3% bovine serum albumin (BSA) in phosphate-buffered saline (PBS) for 1 h at room temperature. Serial dilutions of mouse serum (1:1000 and 1:2000) were added to the wells and incubated for 1–2 h at room temperature to enable specific antibody binding. After washing, horse radish peroxidase (HRP)-conjugated anti-mouse IgG secondary antibody was added and incubated for 1 h at room temperature. Subsequent washes were performed before the addition of tetramethylbenzidine (TMB) substrate in the dark for 10–15 min. The reaction was terminated by 1 N sulfuric acid, and absorbance was measured at 450 nm using an ELISA plate reader to quantify antibody binding [21].

### Collection and preparation of spleenocytes

Immunized mice were anesthetized with isoflurane and then sacrificed through cervical dislocation. The abdominal cavity was opened and the spleen was removed carefully and immediately washed with Dulbecco’s Phosphate Buffered Saline (DPBS). The excised spleen was then placed in a sterile Petri dish filled with Modified Eagle’s Medium (MEM) (without serum) to remove any remaining connective or adipose tissue. The tissue was minced into smaller fragments with scissors under sterile conditions to aid the dispersion of spleenocytes into the medium. The cell suspension obtained was then placed in a sterile centrifuge tube and left to settle for 10–15 min. Following verification of complete sedimentation of the tissue, the supernatant was gently aspirated and plated into a new culture flask. The cells were then incubated and observed for growth during the subsequent week [22].

### Fusion using 50% PEG

#### Preparation of 50% polyethylene glycol (PEG)

PEG 400 was sterilized at 121 °C and cooled to 40 °C under aseptic conditions. The sterilized PEG was then blended with MEM in a 1:1 proportion (5 mL PEG + 5 mL MEM) to obtain a 50% PEG solution. The ready PEG solution was stored at 4 °C and thawed at 37 °C before use on the day of fusion [23].

#### Preparation of 1X HAT medium

A stock solution of 50X HAT medium was prepared by adding the available powdered HAT medium into 10 mL of DMEM. A working solution of 1X HAT medium was made by transferring 1 mL of this stock into a sterile centrifuge tube and adjusting the final volume to 10 mL using DMEM [23].

#### Cell fusion procedure

On fusion day, the thawed PEG solution was equilibrated at 37 °C for 5–7 min. Myeloma cells were centrifuged twice and washed in serum-free growth medium at 5000 rpm for 5 min. The cell pellet was suspended in 5 mL of growth medium. Spleenocytes were mixed with myeloma cells in a ratio of 1:4 in a 15 mL centrifuge tube. The mixed cell suspension was centrifuged at 5000 rpm for 5 min, and the supernatant was discarded and the pellet was resuspended lightly by tapping the tube.

Pre-warmed 50% PEG solution was added dropwise over 1 min with constant agitation of the tube to achieve proper mixing as well as to avoid osmotic shock. For the following 4–5 min, DMEM was slowly added to suspension to reach a final volume of 10 mL, gently and continuously mixing by repeated pipetting. The suspension of cells was centrifuged at 4000 rpm for 10 min, and the supernatant was removed. The pellet was resuspended in 1X HAT medium with 20% FBS. The fused cell suspension that was fused was plated in a 96-well plate and cultured under routine cell culture conditions [23].

### Solid-lipid nanoparticle (SLN) formulation

#### Preparation of carboplatin-Loaded SLNs

Carboplatin-loaded SLNs were prepared by the microemulsion method. The lipid phase, which included soya lecithin, stearic acid, glyceryl monostearate (solid-solid lipid phase), and Pluronic F-127, and the aqueous phase, consisting of distilled water, were separately weighed and heated at 70 °C. The API was added to the lipid phase and agitated for 5 min continuously to ensure uniform dispersion.

The lipid phase was slowly added to the aqueous phase under high-speed homogenization in an ice bath or cold water to produce a stable microemulsion. This emulsion was agitated at 3000 rpm for 2 h. The mixture was filtered through a 0.22-µm membrane filter and diluted to 10 mL using deionized water at a 1:10 ratio in a 10-mL vial. Instantaneous cooling helped in the conversion of nano-droplets from the liquid to solid phase, creating a stable dispersion of SLN. The obtained dispersion was stirred for another 3 h continuously.

The zeta potential and particle size of the formulation were determined using an Anton Paar Litesizer with Kalliope software for analysis. The SLN aqueous suspension was centrifuged at 10,000 rpm for 2 h to separate the nanoparticles. The nanoparticles were blended together with a 20% mannitol solution and freeze-dried. Freeze-dried samples were stored at −89 °C for 24 h [24, 25].

### Characterization of SLN particles

#### Particle size and polydispersity index (PDI)

Prior to size distribution analysis, the dispersed SLN formulation was diluted to a 1:10 ratio in Millipore water. The solution was subsequently filtered using a 0.22-µm membrane filter as described earlier. An Anton Paar Litesizer 500 was used to determine both the particle size (PS) and polydispersity index (PDI) [26].

#### Zeta potential analysis

The zeta potential was analyzed to evaluate the surface charge characteristics of the nanoparticles and to estimate their stability in dispersion.The same diluted sample was used for the determination of the zeta potential. The sample was positioned in a cuvette with electrodes at either end, tightly sealed with a cap, and measured using Kalliope software from Anton Paar [26].

### In vitro cytotoxicity testing using OSCC cell line

#### Cell culture

Human Oral Squamous Cell Carcinoma (OSCC) (NCCS, Pune, India) cells were grown in Dulbecco’s Modified Eagle Medium/Nutrient Mixture F-12 (DMEM/F-12) media supplemented with 10% fetal bovine serum (FBS), 1% penicillin-streptomycin (100 U/mL and 100 µg/mL, respectively), and 400 ng/mL hydrocortisone, as recommended for standard OSCC cell culture. The cells were incubated in a humidified environment containing 5% CO₂ at 37 °C and subcultured at 70–80% confluence using 0.25% trypsin-EDTA.

#### Preparation of test samples

Blank SLNs and Carboplatin-loaded SLNs were dispersed in sterile PBS that was sterile and filtered through a 0.22 μm syringe filter. A free carboplatin solution was prepared in PBS as the control reference. Working concentrations (5, 10, 25, 50, and 100 µg/mL) were prepared fresh from stock solutions prior to each experiment.

#### MTT cytotoxicity assay

The cytotoxic activity of the Carboplatin-loaded SLNs was analyzed with the MTT [3-(4,5-dimethylthiazol-2-yl)−2,5-diphenyltetrazolium bromide] assay. The KB cells were plated at a density of 5 × 10³ cells/well in 96-well plates and incubated overnight.

After 24 h, the medium was removed and replenished with fresh medium supplemented with varying concentrations of carboplatin-loaded SLNs, free carboplatin, blank SLNs, and an untreated control (media only).The treated cells were incubated for 24, 48, and 72 h. For every time point, 20 µL of MTT solution (5 mg/mL in PBS) was added to all the wells and incubated for 4 h at 37 °C. The medium was subsequently removed with care, and 150 µL of dimethyl sulfoxide (DMSO) was added to all the wells to dissolve the formazan crystals produced by metabolically active cells. The absorbance was measured at 570 nm with a microplate reader (Bio-Rad, USA) [27].

#### Sulforhodamine B (SRB) assay

KB cells (1 × 10⁵ cells/ml) were seeded in 96-well plates and left for 24 h of incubation at 37 °C with 5% CO₂. After drug treatment for 72 h, cells were fixed, SRB dye stained, and the absorbance was read at 540 nm to determine growth inhibition [28].

#### Dye exclusion technique

Cell viability was determined by the Trypan Blue dye exclusion technique. Equal volumes of KB cells and 0.4% Trypan Blue solution were combined and kept at room temperature for 3–5 min. A small amount of the mixture was placed on a hemocytometer, and the cells were viewed under a light microscope. Viable cells did not stain, whereas non-viable cells stained blue. Percentage of viable cells was determined from the ratio of unstained cells to total cells [29].

### Statistical analysis

All the experiments were conducted in triplicates to make the results reproducible and reliable. Statistical analysis was performed using appropriate statistical software to determine the significance of the data. Results were reported as mean ± SD, and p-values < 0.05 were regarded as statistically significant.

## Results

### Fluoroscence assay

**Fig. 1:**
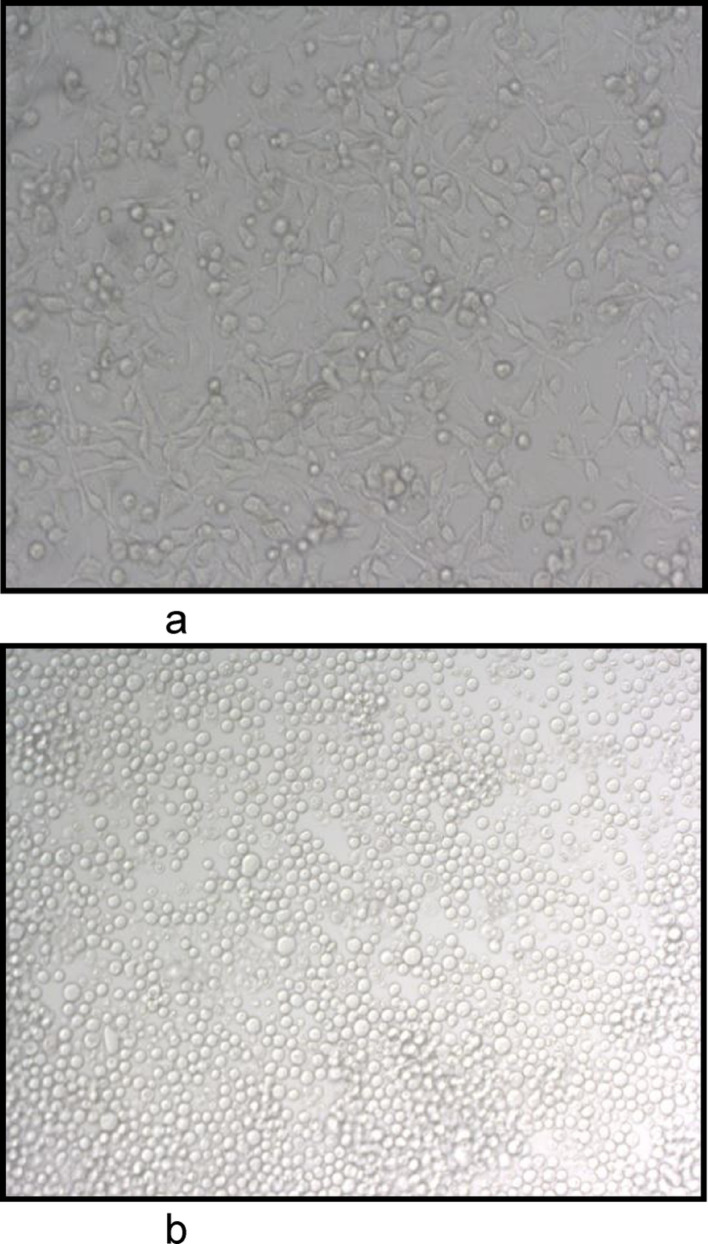
**a** Phase contrast microscopic image of KB cells (Human) (10X). **b** Phase contrast microscopic image of Sp2/01 (Myeloma cells) (10X)

As shown in Fig. 1a and b, KB oral carcinoma cells and Sp2/01 cells were cultured. After staining with a 1:1 acridine orange-ethidium bromide combination, the KB cells exhbited clear spectral properties under the fluorescence microscope (Supplementary Fig. 1a &1b). Most of the cells exhibited green to orange-red fluorescence and were viable. Around 90% of the stained cells were viable on examination as they could incorporated acridine orange. Acridine orange, an intercalating agent, enters both viable and non-viable cells and fluoresces green when it binds to double-stranded DNA (dsDNA) and red when it binds to single-stranded DNA (ssDNA) or RNA. Ethidium bromide, on the other hand, is excluded by intact cell membranes and only stains non-viable cells, fluorescing red. The predominance of green to orange-red fluorescing cells indicated a high ratio of viable KB cells throughout the experiment.

### Immunization and evaluation of serum antibody response

After immunization, the animals were marked with head and tail markings and separated into immunized and unimmunized groups individually (Supplementary Fig. 2a). Two female Balb/c mice in the immunized group were injected intraperitoneally with 0.4 mL KB cell emulsion (1 × 10⁶ cells per dose) on days 0, 6, 13, 20, and 26 (Supplementary Fig. 2b). Both groups’ body weights were measured before and after every injection, as shown in Table 1, to track physiological reactions. During the experiment, both immunized and unimmunized mice gained overall body weight. However, the immunized mice gained comparatively less weight than the unimmunized controls, indicating a potential physiological reaction to the immunization treatment.

To assess the humoral immune response elicited after immunization with KB cells, the serum antibody response was determined after the last booster shot. Seventy-two hours after the final booster dose, approximately 200 µL of blood was collected from immunized mice via the retro-orbital route and transferred into Eppendorf tubes. The samples were allowed to clot, and serum was separated by centrifugation (Supplementary Fig. 3). The collected serum was screened for the presence of antigen-specific antibodies using an enzyme-linked immunosorbent assay (ELISA).

KB cells were fixed in a 96-well plate with 0.05% glutaraldehyde and blocked with 3% bovine serum albumin (BSA) to avoid nonspecific binding. Mouse serum dilutions (1:1000 and 1:2000) were incubated with the fixed cells. The wells were washed followed by treatment with horseradish peroxidase (HRP)-conjugated anti-mouse IgG. The TMB substrate was used to develop the signal, and the absorbance was measured after termination of the reaction by the addition of 1 N sulfuric acid (H₂SO₄) in an ELISA plate reader.

The antibody titer test quantifies the number of specific antibodies present in the serum. Increased titers indicate a successful immune response because of past antigen exposure via vaccination. In this research, wells incubated with secondary antibody and serum exhibited noticeably higher values of absorbance when compared with serum-free control wells, suggesting the presence of specific antigen antibodies. Checkerboard titration established that both the initial immunization and booster injections raised an adequate immune response, as indicated by good and specific antibody binding. Table 2 shows the absorbance values for the serum samples at different serum dilutions (1:2000 and 1:1000).

The absorbance was greater at the 1:1000 dilution, reflecting increased antibody binding, than with the 1:2000 dilution. Average absorbance for each dilution group and the control wells indicates the presence of antigen-specific antibodies at both serum levels, with a weakly increased response at the 1:1000 dilution. From these overall results, it is evident that the immune response was successfully elicited against KB cells.

### Physiological response to KB cell immunization in mice

Changes in body weight in immunized and unimmunized mice were measured over five dosage periods to determine the physiological response of repeated KB cell injections. The body weights at Day 0 (baseline) for Immunized Mouse 1 and Immunized Mouse 2 were 28.2 g and 26.4 g, respectively, `while the unimmunized mouse weighed 26 g. After the first dose, there was a slight gain in body weight in the immunized mice (29.02 g and 27 g), with the unimmunized mouse having little change (26.02 g). With each subsequent dose, immunized mice body weights kept increasing steadily: Immunized Mouse 1 weighed 33.6 g after the third dose and slightly decreased to 33.2 g after the last dose; Immunized Mouse 2 similarly demonstrated steady weight gain, weighing 31.8 g by the end of the study. Conversely, the Unimmunized Mouse attained only marginal weight gain and maintained a plateau at 28.2 g at the last time point. This gradual increase in body weight, especially in the immunized group, might indicate physiological adaptation to immunization and antigen processing.

### Effect of immunization on spleen characteristics and spleenocyte viability

Spleens were harvested from immunized and unimmunized mice after sacrifice and examined grossly (Supplementary Fig. 4). The spleens of immunized mice looked considerably enlarged compared to those of unimmunized controls. A colour change in the immunized spleens was also seen, which shifted from the usual red colour to a brownish colour. These morphological changes suggest physiological alterations induced by immunization, indicating activation of the immune response and antibody production.

After tissue processing, spleenocytes were cultured and isolated. The cells cultured were observed for seven days. An increase in cell density was seen over the period, with spleenocytes being confluent and healthy throughout the incubation time.

### Cell fusion and hybridoma generation

The cell fusion between spleenocytes of immunized mice and myeloma cells was carried out successfully with 50% polyethylene glycol (PEG) as the fusogen. The cells were first plated at a concentration of 100 µL/well in a 96-well plate and cultured under routine culture conditions in HAT medium to favor successful hybridoma production.

Three separate experiments involving cell fusion were performed:

- Trial 1 involved the employment of a greater-than-optimal concentration of HAT, which caused acidification of the medium and, in turn, cytotoxicity.
- Trial 2 was lost with the utilization of an unsuitable medium for spleenocyte isolation, leading to excessive cell lysis and inadequacy of fusion.
- Trial 3 was performed out optimally, resulting in successful fusion and recovery of viable cells. Medium changes were done every 72 h in HAT medium. On the sixth day post-fusion, viable fused cells were evident under the microscope, which shows successful hybridoma formation.

But when HAT medium was replaced by HT medium in later maintenance, a premature cell death was observed, preventing further hybridoma propagation. This finding supports the essential need for HAT in post-fusion viability in early culture.

### Characterization of carboplatin-loaded solid-lipid nanoparticles (SLNs)

Four batches of Carboplatin-loaded SLNs were prepared by the microemulsion method and were each tested to ensure optimal particle size and surface charge. The formulations were reproducible and stable, and the optimized batch was subjected to extensive physicochemical characterization.

#### Zeta Potential

**Fig. 2:**
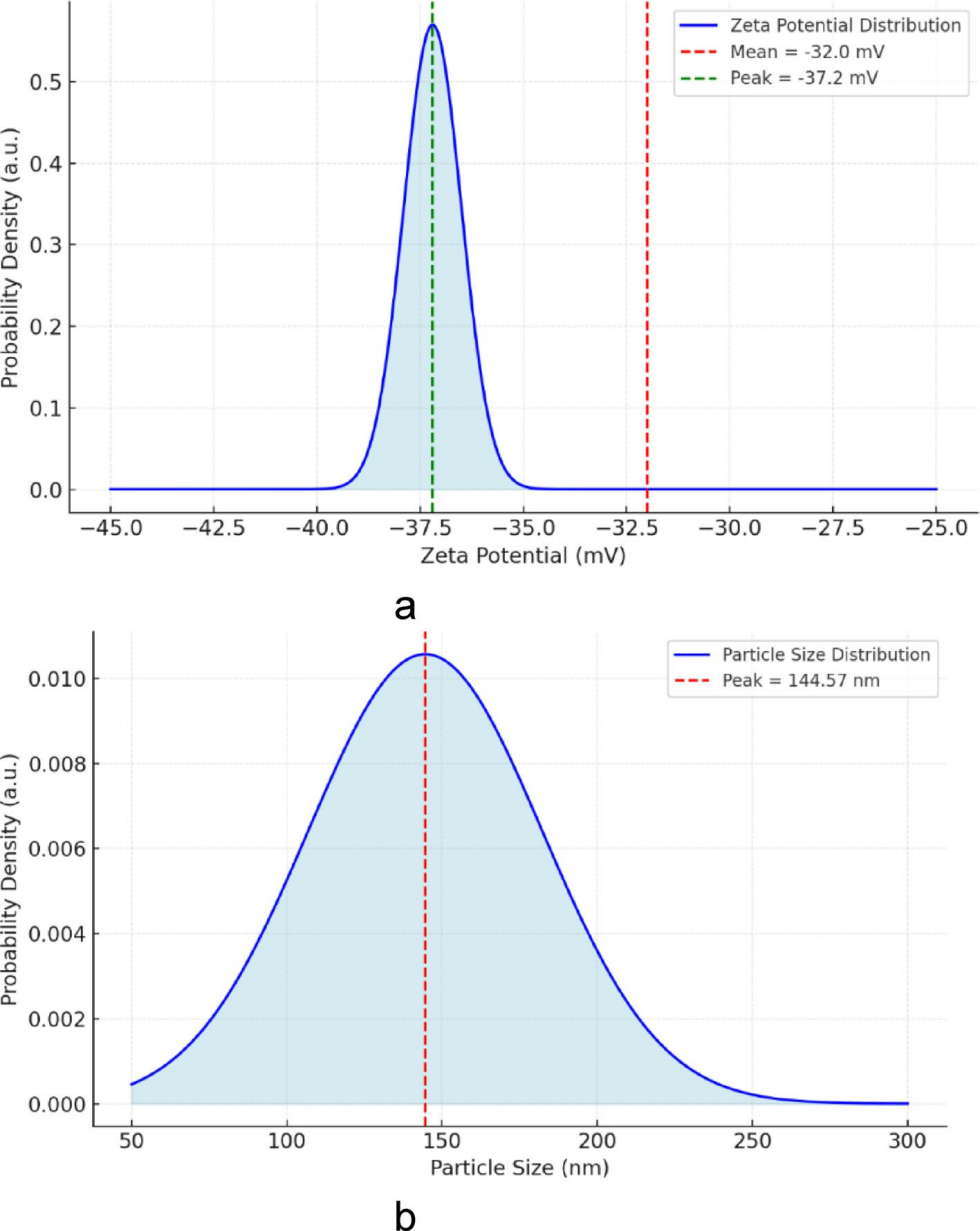
Characterization of Carboplatin-Loaded Soild Lipid Nnaoparticles. **a** Zeta Potential Distribution Curve. **b** Particle size distribution

The zeta potential measurement yielded a mean of − 32.0 mV with a standard deviation of ± 0.7 mV, representing a relatively stable colloidal system (Fig. 2a). The peak of the distribution was at − 37.2 mV, which identifies the prevailing charge population in the sample. The measurement yielded a mean intensity of 593.5 kcounts/s and an optical density of the filter of 3.3029, which expresses the scattering nature of the sample. In addition, the conductivity was 0.008 mS/cm, while the electrophoretic mobility was − 1.6646 μm·cm/V·s, reinforcing the negative surface charge of the particles. The sample was highly transparent with a transmittance of 91.5%, implying no aggregation or particle clumping.

#### Particle Size and Polydispersity Index (PDI)

The dynamic light scattering (DLS) study provided a hydrodynamic diameter of 144.57 nm, which represented the average particle size in the sample (Fig. 2b). The system had a polydispersity index (PDI) of 26.1%, representing moderate size distribution with some heterogeneity between the particles. The measurement indicated a mean intensity of 294.0 kcounts/s and an absolute intensity of 2056.2 kcounts/s, representing high light scattering properties. Diffusion coefficient was found to be 3.4 μm²/s, and the intercept g1² value was 0.9279, reflecting the quality of the data. The sample was of high clarity and transmittance of 92.3%, and the baseline value was 1.051, reflecting the reliability of the measurements.

In total, the characterization data validate effective formulation of Carboplatin-loaded SLNs with optimal size, charge, and stability properties, which are appropriate for subsequent drug delivery investigation.

### Cytotoxicity of carboplatin-loaded SLNs on OSCC cells

**Fig. 3:**
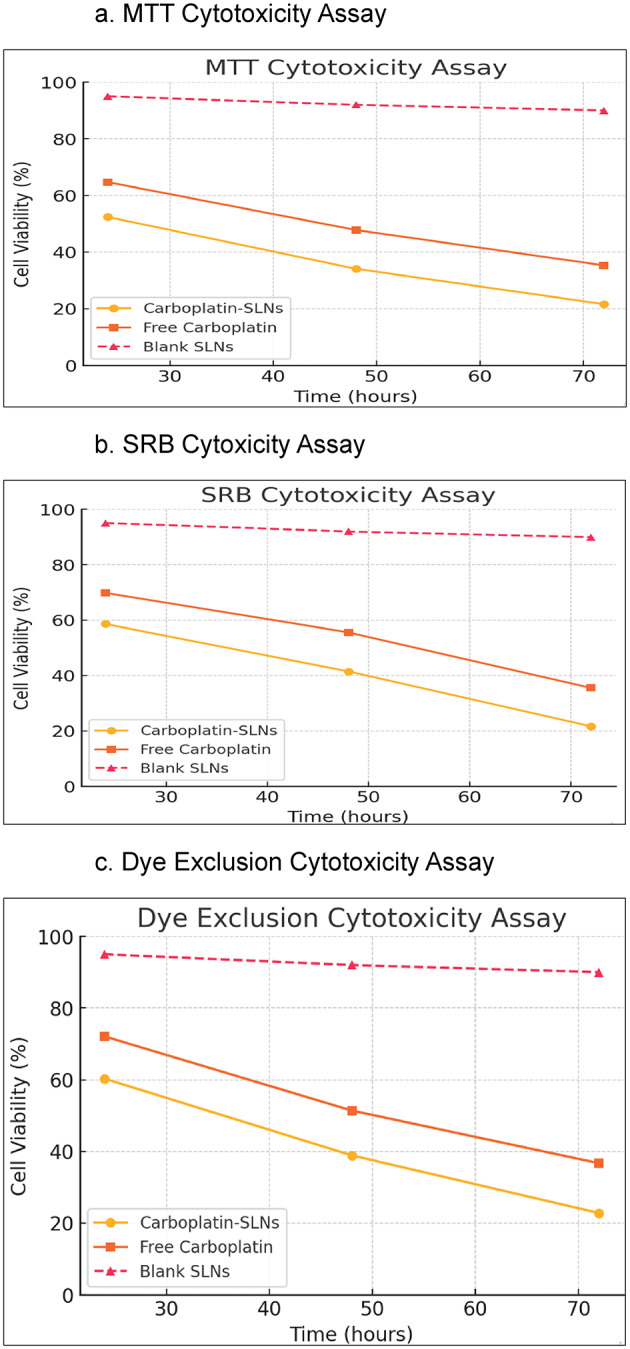
Comparative Cytotoxicity Analysis of Carboplatin-Loaded SLNs on OSCC Cells. **a** MTT Cytotoxicity Assay. **b** SRB Cytoxicity Assay. **c** Dye Exclusion Cytotoxicity Assay

The SLNs-loaded Carboplatin cytotoxicity was always greater than free Carboplatin in all assays (MTT, SRB, and Dye Exclusion), with a definite time-dependent decrease in cell viability at 24, 48, and 72 h. In the MTT assay, viability dropped from 52.4% at 24 h to 21.6% at 72 h with SLNs, whereas for free Carboplatin it was 64.7% and 35.3% (Fig. 3a). In the same way, SRB assay revealed viability from 58.6% to 21.6% for SLNs, compared to 69.8% to 35.5% for free drug, which demonstrates higher protein binding and cellular retention (Fig. 3b). Dye Exclusion assay also supported the data with SLNs decreasing viability to 22.8% from 60.3%, compared to 72.1% to 36.7% for free Carboplatin (Fig. 3c). In all the assays, Blank SLNs showed minimal toxicity with > 90% cell viability, validating the biocompatibility of the lipid carrier. These findings collectively show that the SLN formulation considerably enhances drug efficacy through improved cellular uptake and sustained release.

## Discussion

The current research was able to successfully integrated immunological methods, nanotechnology, and cancer cell biology to assess the immunogenicity and therapeutic efficacy of Carboplatin-loaded solid-lipid nanoparticles (SLNs) for the treatment of oral squamous cell carcinoma (OSCC). The results cumulatively provide information regarding both antibody production and cytotoxicity-based treatment approaches.

KB oral cancer cells were cultured under standard in vitro conditions using MEM supplemeneted with 10% FBS. Subculturing at 80–90% confluency ensure maintainance of healthy proliferative state, and the viability of the cells was ascertained by fluorescence staining using acridine orange and ethidium bromide, which indicated > 90% viability confirming suitability of the culture for further experiments. The differential staining involving viable cells emitting green fluorescence (AO) and non-viable cells emitting red fluorescence (EtBr) ensured membrane integrity and integrity of nucleic acids in most of the population [30].

Intraperitoneal delivery of KB cell emulsions into Balb/c mice provoked a physiological reaction observable by sustained increases in body weight throughout the immunized cohort. This gradual weight gain may reflect immune activation resulting from repeated antigenic stimulation. The antibody screening utilizing ELISA showed clear evidence of a strong humoral response [31]. Absorbance readings were increased for serum dilutions (1:1000 and 1:2000) than in controls, establishing that the immunized mice contained KB cell-specific antibodies. This is an affirmation that the antigenic preparation was effective in developing a strong and specific immune response, in accordance with previous findings of tumor-derived immunizations inducing systemic immunity.

Analysis after sacrifice indicated that immunized mice spleens had undergone hypertrophy and color change, indicating active immune proliferation. The successful isolation and culture of live spleenocytes for up to 7 days further testified to continued immunological activity. PEG-400-mediated fusion of myeloma cells with spleenocytes led to hybridoma formation. Whereas Trials 1 and 2 were unsuccessful because of medium-related and concentration-related mistakes, the optimized conditions of Trial 3 produced viable fusion products, as indicated by cell proliferation in HAT medium. Importantly, a shift from HAT to HT medium caused cell death, highlighting the critical role of selective pressure in initial hybridoma stabilization.

Nanoparticle-mediated drug delivery was attained by the method of microemulsion. The SLNs optimized had a mean particle size of 144.57 nm, within the favorable nanometric range for cellular entry. A polydispersity index (PDI) value of 0.261 showed a relatively monodisperse formulation. The zeta potential value of −32 mV implies high colloidal stability through electrostatic repulsion. Other parameters like high transmittance (92.3%) and suitable diffusion coefficient validate the physical stability and dispersion stability of the nanoparticles. These characteristics together render the SLNs good candidates for drug delivery in cancer treatment.The stability of the optimized SLN formulation was evaluated by monitoring particle size and zeta potential over a 30-day period at room temperature. No significant variations were observed in either parameter up to 30 days, confirming the physical stability of the SLN dispersion during storage.

The relative cytotoxicity information of MTT, SRB, and Dye Exclusion assays evidently illustrates that Carboplatin-loaded SLNs have greater anticancer efficacy against KB cells than free Carboplatin. For all the time points (24, 48, and 72 h), the SLN formulation demonstrated an enhanced decrease in cell viability, signifying greater drug delivery and prolonged intracellular release. The enhanced cytotoxicity of the SLNs as evidenced by viability values decreasing to 21–22% at 72 h versus 35–37% for the free drug implies enhanced cellular uptake based on the nano-sized lipid carrier. The results of the SRB assay also corroborate this finding by indicating greater drug-protein interactions and inhibition of growth and the Dye Exclusion assay verifies greater percentages of non-viable cells following treatment with SLN. The increased efficacy of the SLN formulation is brought about by its capacity to enhance Carboplatin’s bioavailability, escape cellular efflux pumps, and provide long intracellular retention of the drug. Notably, the low cytotoxicity of Blank SLNs (all assays had > 90% cell viability) highlights the high biocompatibility of the lipid carrier system, which renders it a safe and efficient drug delivery platform. Overall, these results highlight the therapeutic capability of Carboplatin-loaded SLNs as a novel therapeutic alternative to standard Carboplatin treatment, with enhanced efficacy and decreased off-target toxicity.

These results not only confirm the effectiveness of SLN-mediated delivery of Carboplatin but also highlight the effective induction of an antigen-specific immune response against KB tumor cells. The two-pronged approach—immunological priming by KB cell vaccination and chemotherapeutic targeting by SLNs—can provide a synergistic effect in oral cancer therapy. In addition, hybridoma technology provides a basis for future monoclonal antibody development against tumor antigens.

**Table 1:** Body weight changes in Balb/C Mice during the procedure

Animal dosage	Immunised mice 1 (wt. In gms.)	Immunised mice 2 (wt. In gms.)	Unimmunised mice (wt. In gms.)
0	28.2	26.4	26
1	29.02	27	26.02
2	31.2	29.6	28
3	33.6	30.2	28
4	33.2	31.8	28.2

**Table 2:** OD values of serum collected from the Immunised Mice Model

Row	1:2000 Ratio	1:1000 Ratio	Control (C/C)
A	0.1533	0.1525	0.1912
B	0.1806	0.1622	0.1886
C	0.1963	0.1668	0.1797
D	0.1645	0.2615	0.1823
E	0.1867	0.1578	0.1807
F	0.1952	0.1997	0.1933
G	0.1747	0.1291	0.2117
H	0.2350	0.1729	0.1986
Average	0.18747	0.1938	0.09434

## Conclusion

The current research work offers a preliminary investigation into the development of immunotherapeutic and nanomedicine-based approaches for the management of OSCC. Immunization of BALB/c mice with antigens derived from KB cells showed the production of antibodies, as evident from ELISA and spleenocyte cytotoxicity studies. Successful fusion of spleenocytes with myeloma cells and the formation of hybridomas suggest the potential feasibility of developing monoclonal antibodies against tumor-specific epitopes. However, further studies are required to validate the specificity of these antibodies. In addition, Carboplatin-encapsulated SLNs have been formulated, and the results showed acceptable physicochemical characteristics. The in vitro cytotoxicity studies using the KB cell line showed the enhanced potential of the SLNs in cancer treatment compared with Carboplatin, as evident from the IC50 value and time-dependent inhibition of cell viability. Overall, the current research article offers preliminary scientific evidence on the potential integration of immunotherapeutic and nanomedicine-based approaches in OSCC treatment. However, the current research article is exploratory in nature, and further studies are required to validate the potential of the current approach.

## Electronic Supplementary Material

Below is the link to the electronic supplementary material.

Supplementary File 1

## Data Availability

Data sharing not applicable to this article as no datasets were generated or analysed during the current study.

## Abbreviations

AO: Acridine orange
BSA: Bovine serum albumin
dsDNA: Double-stranded DNA
DLS: Dynamic light scattering
DMSO: Dimethyl sulfoxide
ELISA: Enzyme-linked immunosorbent assay
EtBr: Ethidium bromide
FBS: Fetal bovine serum
HAT: Hypoxanthine-aminopterin-thymidine
HRP: Horseradish peroxidase
HT: Hypoxanthine-thymidine
KB cells: Oral carcinoma cell line
MEM: Minimum essential medium
OSCC: Oral squamous cell carcinoma
PBS: Phosphate-buffered saline
PDI: Polydispersity index
PEG: Polyethylene glycol
SD: Standard deviation
SLNs: Solid-lipid nanoparticles
SRB: Sulforhodamine B
ssDNA: Single-stranded DNA
TMB: 3,3′,5,5′-Tetramethylbenzidine

## References

[1] Nethan ST, Ravi P, Gupta PC. Epidemiology of oral squamous cell carcinoma in Indian scenario. InMicrobes and oral squamous cell carcinoma: A Network spanning infection and inflammation 2022. (pp. 1–7). Singapore: Springer Nature Singapore.

[2] Tran Q, Maddineni S, Arnaud EH, Divi V, Megwalu UC, Topf MC, Sunwoo JB. Oral cavity cancer in young, non-smoking, and non-drinking patients: A contemporary review. Crit Rev Oncol/Hematol. 2023;190:104112. 37633348 10.1016/j.critrevonc.2023.104112 PMC10530437

[3] Asthana S, Labani S, Kailash U, Sinha DN, Mehrotra R. Association of smokeless tobacco use and oral cancer: a systematic global review and meta-analysis. Nicotine Tob Res. 2019;21(9):1162–71. 29790998 10.1093/ntr/nty074

[4] Kavarthapu A, Gurumoorthy K. Linking chronic periodontitis and oral cancer: A review. Oral Oncol. 2021;121:105375. 34140233 10.1016/j.oraloncology.2021.105375

[5] Yang L, Shi P, Zhao G, Xu J, Peng W, Zhang J, Zhang G, Wang X, Dong Z, Chen F, Cui H. Targeting cancer stem cell pathways for cancer therapy. Signal Transduct Target therapy. 2020;5(1):8. 10.1038/s41392-020-0110-5 PMC7005297 32296030

[6] Sasikumar PG, Ramachandra M. Small-molecule immune checkpoint inhibitors targeting PD-1/PD-L1 and other emerging checkpoint pathways. BioDrugs. 2018;32(5):481–97. 30168070 10.1007/s40259-018-0303-4

[7] Lee L, Gupta M, Sahasranaman S. Immune checkpoint inhibitors: An introduction to the next-generation cancer immunotherapy. J Clin Pharmacol. 2016;56(2):157–69. 26183909 10.1002/jcph.591

[8] Ghemrawi R, Abuamer L, Kremesh S, Hussien G, Ahmed R, Mousa W, et al. Revolutionizing cancer treatment: Recent advances in immunotherapy. Biomedicines. 2024;12(9):2158. 39335671 10.3390/biomedicines12092158 PMC11429153

[9] Zahavi D, Weiner L. Monoclonal antibodies in cancer therapy. Antibodies. 2020;9(3):34. 32698317 10.3390/antib9030034 PMC7551545

[10] Modjtahedi H, Ali S, Essapen S. Therapeutic application of monoclonal antibodies in cancer: advances and challenges. Br Med Bull. 2012;104(1):41–59. 23118261 10.1093/bmb/lds032

[11] Jefferis R. Recombinant proteins and monoclonal antibodies. Adv Glycol Biotechnol. 2017;281–318. 10.1007/10_2017_32 29071407

[12] Ponziani S, Di Vittorio G, Pitari G, Cimini AM, Ardini M, Gentile R, Iacobelli S, Sala G, Capone E, Flavell DJ, Ippoliti R. Antibody-drug conjugates: the new frontier of chemotherapy. Int J Mol Sci. 2020;21(15):5510. 32752132 10.3390/ijms21155510 PMC7432430

[13] Manzari MT, Shamay Y, Kiguchi H, Rosen N, Scaltriti M, Heller DA. Targeted drug delivery strategies for precision medicines. Nat Rev Mater. 2021;6(4):351–70. 34950512 10.1038/s41578-020-00269-6 PMC8691416

[14] Alsaad AA, Hussien AA, Gareeb MM. Solid lipid nanoparticles (SLN) as a novel drug delivery system: A theoretical review. Syst Rev Pharm. 2020;11(5):259–73.

[15] Arana L, Gallego L, Alkorta I. Incorporation of antibiotics into solid lipid nanoparticles: a promising approach to reduce antibiotic resistance emergence. Nanomaterials. 2021;11(5):1251. 34068834 10.3390/nano11051251 PMC8151913

[16] Madkhali OA. Perspectives and prospective on solid lipid nanoparticles as drug delivery systems. Molecules. 2022;27(5):1543. 35268643 10.3390/molecules27051543 PMC8911793

[17] Werengowska-Ciećwierz K, Wiśniewski M, Terzyk AP, Furmaniak S. The chemistry of bioconjugation in nanoparticles-based drug delivery system. Adv Condens Matter Phys. 2015;2015(1):198175.

[18] Abdelhamid MS, Wadan AH, Saad HA, El-Dakroury WA, Hageen AW, Mohammed DH, Mourad S, Mohammed OA, Abdel-Reheim MA, Doghish AS. Nanoparticle innovations in targeted cancer therapy: advancements in antibody–drug conjugates. Naunyn-Schmiedeberg’s Arch Pharmacol. 2025;18:1–21. 10.1007/s00210-024-03764-7 39825965

[19] An WF. Fluorescence-based assays. InCell-based assays for high-throughput screening: methods and protocols. 2009. (pp. 97–107). Totowa, NJ: Humana.

[20] Li F, Vijayasankaran N, Shen A, Kiss R, Amanullah A. Cell culture processes for monoclonal antibody production. InMAbs 2010. (Vol. 2, No. 5, pp. 466–79). Taylor & Francis. 10.4161/mabs.2.5.12720 PMC2958569 20622510

[21] Eskinazi DP, Molinaro GA, Abemayor E, Martin SE, Zighelboim J. Monoclonal antibodies against squamous cell carcinoma. Oral Surg oral Med oral Pathol. 1985;60(4):377–81. 10.1016/0030-4220(85)90259-2 3903597

[22] Yokoyama WM, Christensen M, Santos GD, Miller D, Ho J, Wu T, et al. Production of monoclonal antibodies. Curr Protoc Immunol. 2013;102(1):2–5. 10.1002/0471142735.im0205s102 24510488

[23] Gefter ML, Margulies DH, Scharff MD. A simple method for polyethylene glycol-promotedhybridizationofmousemyelomacells. Somat Cell Mol Genet. 1977;3(2):231–6. 10.1007/BF01551818 605383

[24] Sandri G, Motta S, Bonferoni MC, Brocca P, Rossi S, Ferrari F, et al. Chitosancoupledsolidlipidnanoparticles:tuningnanostructureandmucoadhesion. Eur J Pharm Biopharm. 2017;110:13–8. 27989765 10.1016/j.ejpb.2016.10.010

[25] Eskiler GG, Cecener G, Dikmen G, Egeli U, Tunca B. Solid lipid nanoparticles: Reversal of tamoxifen resistance in breast cancer. Eur J Pharm Sci. 2018;120:73–88. 10.1016/j.ejps.2018.04.040 29719240

[26] Jagani HV, Josyula VR, Palanimuthu VR, Hariharapura RC, Gang SS. Improvement oftherapeutic efficacy of PLGA nanoformulation of siRNA targeting anti-apoptotic Bcl-2throughchitosan coating. Eur J Pharm Sci. 2013;48(4–5):611–8. 23291045 10.1016/j.ejps.2012.12.017

[27] Ghasemi M, Liang S, Luu QM, Kempson I. The MTT assay: a method for error minimization and interpretation in measuring cytotoxicity and estimating cell viability. InCell viability assays: Methods and protocols 2023. (pp. 15–33). New York: Springer US. 10.1007/978-1-0716-3052-5_2 37142913

[28] Shakil MS, Rana Z, Hanif M, Rosengren RJ. Key considerations when using the sulforhodamine B assay for screening novel anticancer agents. Anticancer Drugs. 2022;33(1):6–10. 34261912 10.1097/CAD.0000000000001131

[29] Patrón-Romero L, Luque PA, Soto-Robles CA, Nava O, Vilchis-Nestor AR, Barajas-Carrillo VW, Martínez-Ramírez CE, Méndez JC, Palacios JA, Ávila ML, Almanza-Reyes H. Synthesis, characterization and cytotoxicity of zinc oxide nanoparticles by green synthesis method. J Drug Deliv Sci Technol. 2020;60:101925.

[30] Vale C, GS R. Toxicological studies with cells. Environmental Toxicology: Non-Bacterial Toxins. 2024:135–70.

[31] Cervenak J, Kurrle R, Kacskovics I. Accelerating antibody discovery using transgenic animals overexpressing the neonatal Fc receptor as a result of augmented humoral immunity. Immunol Rev. 2015;268(1):269–87. 26497527 10.1111/imr.12364

