# Supplementary material for: Production of monoclonal antibodies (mAbs) against oralcarcinoma & its bioconjugation with solid lipid nanoparticles (SLN) for drug delivery
Source: Discov Oncol. 2026 May 26;17:1061. doi: 10.1007/s12672-026-05229-0 (PMC13391474; doi:10.1007/s12672-026-05229-0)

Supplementary Figure 1a : Glass slides with KB cells on coverslip stained with Achridine orange and Ethididium Bromide


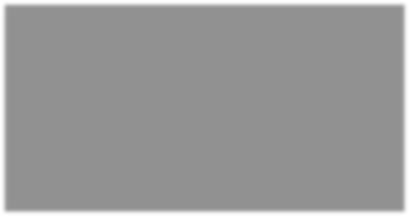

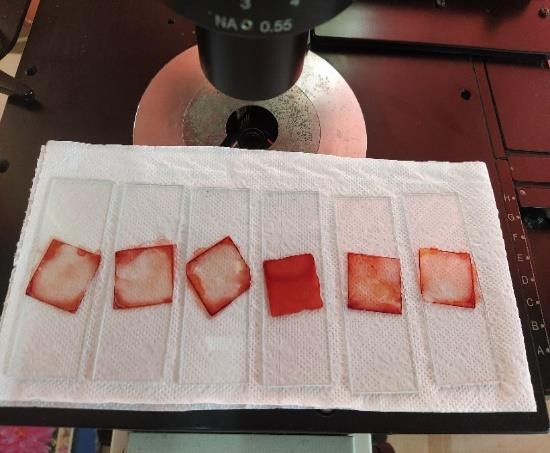


Supplementary Figure 1b : Microscopic image of cell staining (10X)


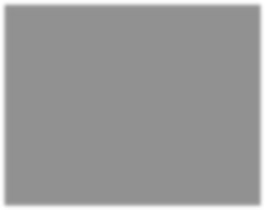

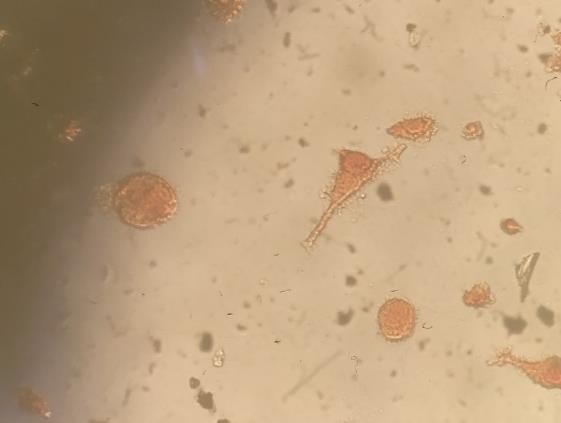

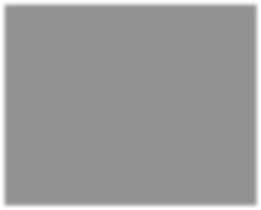

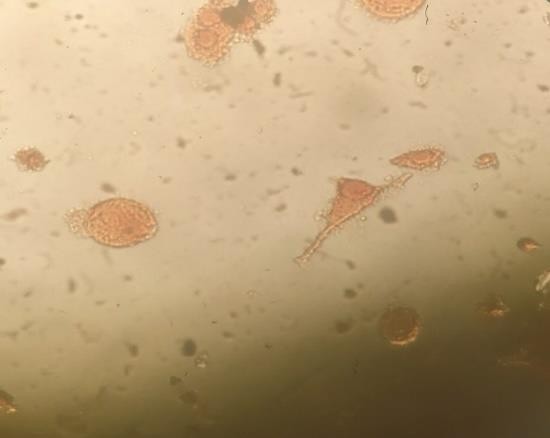


Supplementary Figure 2a. Labelled femalemiceforImmunisation (a) Labelled head marked mice for immunisation,(b) Unlabelledmicenoimmunisation asacontrol


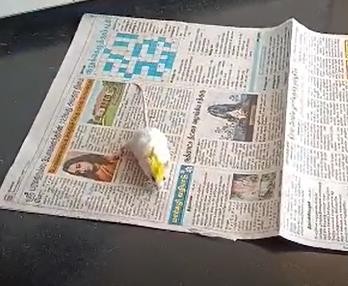

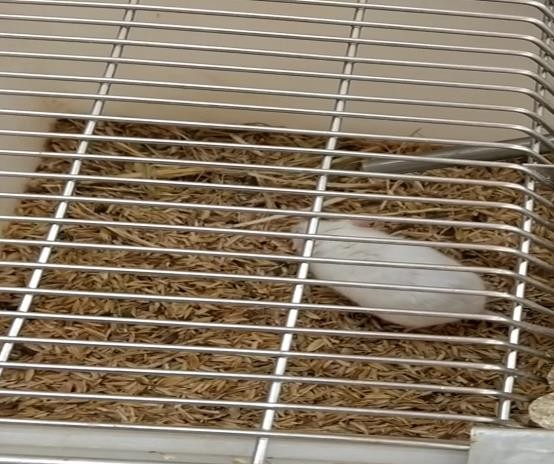


Supplementary Figure 2b. Immunisation of Balb/C female mice with KB cell in PBS and Freund’s Adjuvant- 0.4ml dose injected to both the mice animal


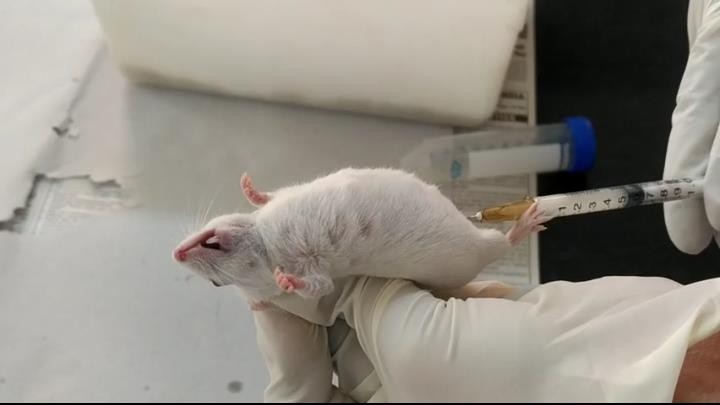

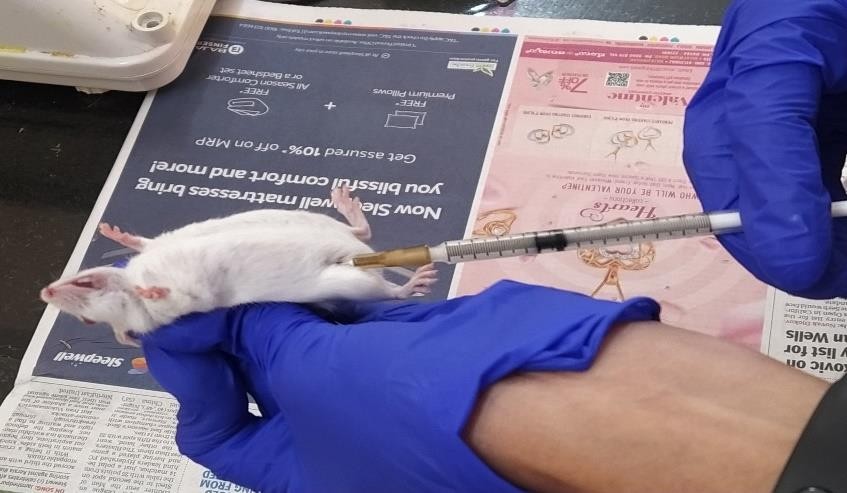


Supplementary Figure 3. Collectionof Bloodfromimmunizedmice


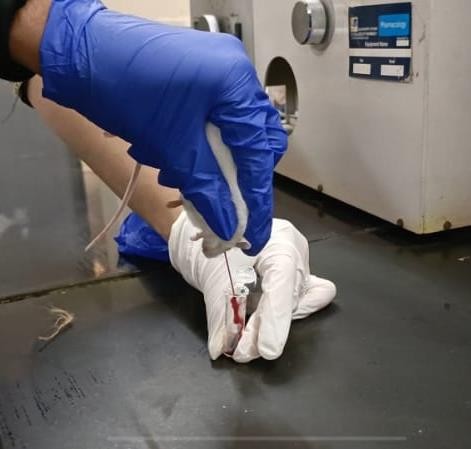

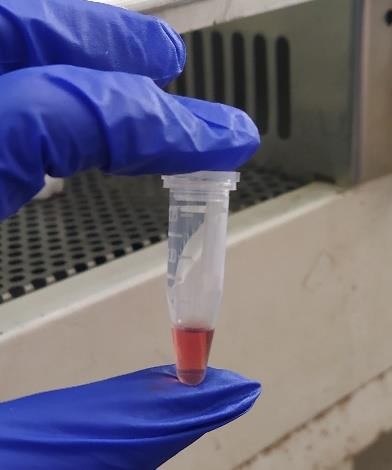


Supplementary Figure 4. Spleenscollectedfrom Unimmunizedandimmunizedmice


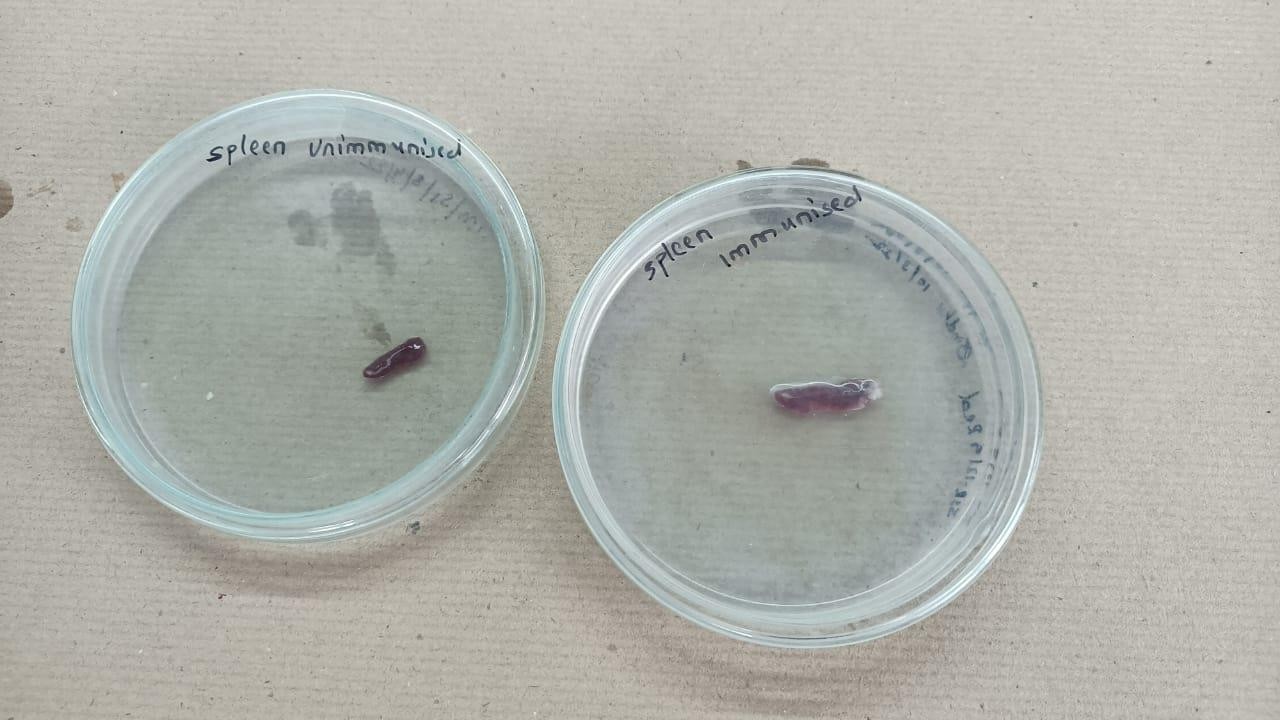

Supplement: Supplementary file 1 [file 12672_2026_5229_MOESM1_ESM.docx]
